# Impact of Early Exposure to Play Materials on Motor Development in High-Risk Infants: A Randomised Controlled Trial

**DOI:** 10.34763/jmotherandchild.20232701.d-22-00025

**Published:** 2023-07-06

**Authors:** Mrunmayi S Gadre, Vinuta R Deshpande

**Affiliations:** Department of Paediatric Physiotherapy, KLE Institute of Physiotherapy, Belagavi, Karnataka, India

**Keywords:** Early intervention, play materials, motor development, high-risk infants

## Abstract

**Background:**

The purpose of this study was to determine the impact of early exposure to play materials on motor development in high-risk infants.

**Materials and methods:**

A 1:1 parallel group randomised control study was conducted. A total of 36 participants were recruited, with 18 in each group. The intervention lasted 6 weeks for both groups, with follow-ups in the 2nd and 4th weeks. The Peabody Developmental Motor Scale 2nd Edition (PDMS-2) was used as an outcome measure. The data was analysed using the Likelihood Ratio test, Chi-square test, independent sample t-test, and paired t-test.

**Results:**

There was no difference between the groups except for the raw reflex scores (t = 3.29, p = 0.002), raw stationary scores (t = 4.26, p < 0.001), standard stationary scores (t = 2.57, p = 0.015), and Gross Motor Quotient (GMQ) (t = 3.275, p = 0.002). Statistical significance within the experimental group was observed in the raw reflex (t = −5.16, p < 0.001), stationary (t = −10.5, p < 0.001), locomotion (t = −5.67, p < 0.001), grasp (t = −4.68, p < 0.001), and visual motor (t = −5.03, p < 0.001) scores, as well as the standard stationary (t = −2.87, p = 0.010), locomotion (t = −3.43, p = 0.003), grasp (t = −3.28, p = 0.004), and visual motor (t = −5.03, p < 0.001) scores. Quotients were the GMQ (t = −7.31, p < 0.001), Total Motor Quotient (TMQ) (t = −5.71, p < 0.001), Fine Motor Quotient (FMQ) (t = −6.48, p < 0.001). Conclusions: The current study concludes that a six-week treatment of early exposure to age-appropriate toys is advantageous in enhancing motor development in high-risk neonates.

## Introduction

The Lancet series on early child development estimates that there are 250 million children under 5 years of age in low- and middle-income countries who are at risk of not achieving their development potential due to issues relating to poverty, malnutrition, infectious diseases, and poor access to healthcare [1]. The prevalence of global neurodevelopmental disturbance is estimated to be around 19.8%, of which 42.5% of children showed a lag in personal social skills, followed by 38.1% who showed a delay in motor skills [2,3]. In India, out of the 27 million babies born every year, 3.5 million are premature [4]. With survival rates of preterm and low-birth-weight infants improving, there is an increase in the number of these infants who face motor impairments later in life, which can range from developmental coordination disorder to cerebral palsy (CP) [5]. This has led to extensive follow-up programmes to determine which of these infants need intervention. Recent studies suggest that intervention may be most effective when it is applied during infancy, when there is rapid myelination and plasticity of the brain [6,7].

In a developing country like India, issues like poverty, low education status, malnutrition, and unstimulating home environments can have detrimental effects on developmental outcomes in young infants [3,8]. In view of this, a range of interventions aimed to promote child development through parent-child interaction activities have been reported to be associated with positive influences on cognitive, language, motor, and behavioural outcomes during early childhood [9,10,11,12,13,14]. Although specific home environment and motor development characteristics have been examined [15,16,17,18], interestingly, the most striking and consistent findings have been that the ‘availability of stimulating play materials were more strongly related to child development status than global measures of environmental quality such as socioeconomic status’ [19]. A low-cost, preventive, parent-oriented primary care program can influence the outcome of childhood neurodisability; however, little evidence exists to determine the effects of play material in preterm infants with low birth weight on early neurodevelopmental outcome. Thus, the present study was conducted with the objective to evaluate the impact of early play exposure to play materials on motor development in high-risk infants using PDMS-2.

## Materials and Methods

A parallel design randomised control trial with an allocation ratio of 1:1 was conducted from March 2021 to April 2022 in Belagavi, Karnataka, South India. Approval for the study was obtained from the KLE Institute of Physiotherapy’s Institutional Ethical Committee (KIPT/Sl no. 579, dated 16 July 2021) and registered with CTRI (CTRI/2022/01/039604). Participants recruited and included were parent-infant dyads, with infants of corrected age (premature baby’s chronological age minus the number of weeks or months they were born early) 5 months 0 days to 6 months 30 days, who were permanent residents of Belgavi and interested in participating as well as willing to provide written informed consent. Infants with a history of or diagnosis with any birth defect or congenital abnormality, or who had undergone any surgery in the previous three months, were excluded. A sample size of 36 was obtained using the formula $\text{n}=\frac{2{\left({\text{z}}_{\text{a}}+{\text{z}}_{\text{b}}\right)}^{2}*{\text{s}}^{\text{2}}}{d^{2}}$ , where Z_α_ = 1.96, σ is the population variance (0.1), and *d* is the difference we would like to detect (0.09). *n* = 2(1.96+1.28)^2^ * 0.1^2^ / (0.09)^2^, with 18 in each group. Randomization occurred using a sealed envelope method with a random allocation sequence. The co-investigator assessing the outcome using PDMS-2 did so blindly.

## Outcome Measure

### Peabody Developmental Motor Scale – 2nd Edition [PDMS-2]

The PDMS-2 assesses fine and gross motor skills of children from birth to 5 years old. The four gross motor subtests are reflex, stationary, locomotion, and object manipulation, while the two fine motor subtests are grasping and visual-motor integration. The total score is determined by the sum of the points of each subtest. The manual reports a good index of internal consistency for each subtest (α = 0.89 to 0.95) and for each motor quotient (0.96 to 0.97), acceptable temporal stability through the test-retest with an interval of one week (α = 0.73 to 0.96 depending on the age level), and high inter-observer fidelity (which varied between 0.97 to 0.99 for subtests and between 0.96 and 0.98 for the motor quotients) [20].

## Intervention

Parent-infant dyads from both groups participated in the intervention for six weeks. All the toys were included in an age-appropriate toy package. The parents in the control group were interviewed about the toys they used to play with their infants, as well as how much time they gave their babies to play with them, and how many times a day the baby liked to play with them. Based on the information presented by the parents, the investigator educated the parents regarding the need for giving toys to the baby on a regular basis for his or her continuous development. The experimental group received a ready-to-use play kit and detailed instructions on how to use the toys. A detailed description of the toys used, games, and targeted motor skill development is provided as a supplementary document. The intervention lasted for six weeks, with at least two to three 20-minute sessions every day. A follow-up assessment to check the adherence of the intervention was done at the second and fourth weeks. To assess post-intervention outcomes, the co-investigator visited the participants’ homes.

## Results

**Figure 1.**
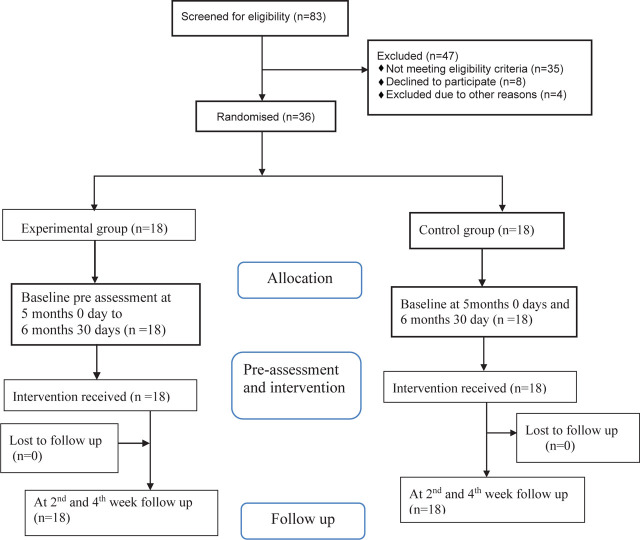
CONSORT flow diagram of progress through the phases of randomization in both groups.

Participants were chosen based on their eligibility. We have provided a flow diagram according to CONSORT, depicting the progression through phases of a parallel group randomised experiment (Fig. 1). Due to one dropout, 35 participants were assessed and analysed, with 18 in the intervention group and 17 in the control group. Gender was one of the dependent variables in the study participants’ characteristics. There was a total of 35 participants, out of which 22 were male, accounting for 61.1% (14 male infants(77%) were in the experimental group and 8 (44.4%) were in the control group). 13 participants were female, which accounted for 38.9% (4 female infants (22.2%) were in the experimental group and 9 (55.6%) were in the control group). There was a significant association based on gender (*p* = 0.040) when applied to the chi square test (Table 1).

**Table 1.** Demographic characteristics of study participants for descriptive and categorical variables

**Variable**	**Categories**	**Frequency %**	**Mean ± SD**
Birth weight (Kg)	-	-	2.22 ± 0.54
Gestational age (Weeks)	-	-	33.89 ± 1.39
Chronological age (Months)	-	-	7.14 ± 0.83
Corrected age (Months)	-	-	5.33 ± 0.48

**Variable**	**Categories**	**Experimental Group n (%)**	**Control Group n (%)**	**Chi-Square/Likelihood Ratio**	**p-value**
Gender	Male	14 (77.8)	8 (44.4)	4.208	0.040^*^
	Female	4 (22.2)	9 (55.6)		
Socioeconomic Status	Lower middle (III)	11 (61.1)	15 (83.3)	4.946#	0.084
	Upper lower (IV)	0 (0)	0 (0)		
	Upper middle (II)	7 (38.9)	2 (11.1)		

SD: standard deviation,

* statistically significant (level of significance: *p* < 0.05)

PDMS-2 gives scores in terms of raw scores, standard scores, gross motor quotient, fine motor quotient and total motor quotient (Table 2). Paired t-test results show that the p- and t-values for raw scores in the experimental group for the reflex (t = −5.16, *p* < 0.001), stationary (t = −10.5, *p* < 0.001), locomotion (t = −5.67, *p* < 0.001), grasp (t = −4.68, *p* < 0.001), and visual motor (t = −5.03, *p* < 0.001) components were statistically significant.

**Table 2.** Comparison of pre- and post-test raw, standard, and quotient scores of PDMS-2 within both groups

**Group**	**PDMS-2 Subtest**	**Paired Differences**				**t**	**df**	**p-value**

		**Mean**	**S.D.**	**95% Confidence Interval of the Difference**				
Experimental	Reflex (Raw Score)	−1.83	1.51	−2.58	−1.09	−5.169	17	< 0.001^*^
	Reflex (Standard Score)	−0.44	0.98	−0.93	0.05	−1.917	17	0.072
	Stationary (Raw Score)	−3.94	1.59	−4.74	−3.15	−10.529	17	< 0.001^*^
	Stationary (Standard Score)	−1.89	2.78	−3.27	−0.50	−2.878	17	0.010^*^
	Locomotion (Raw Score)	−5.72	4.28	−7.85	−3.59	−5.67	17	< 0.001^*^
	Locomotion (Standard Score)	−1.06	1.31	−1.70	−0.41	−3.432	17	0.003^*^
	Grasping (Raw Score)	−5.39	4.88	−7.81	−2.96	−4.688	17	< 0.001^*^
	Grasping (Standard Score)	−0.78	1.00	−1.28	−0.28	−3.289	17	0.004^*^
	Visual Motor Integration (Raw Score)	−5.44	4.59	−7.73	−3.16	−5.03	17	< 0.001^*^
	Visual Motor Integration (Standard Score)	−0.89	0.83	−1.30	−0.48	−4.531	17	< 0.001^*^
	GMQ	−2.89	1.68	−3.72	−2.06	−7.311	17	< 0.001^*^
	FMQ	−2.11	1.57	−2.89	−1.33	−5.713	17	< 0.001^*^
	TMQ	−4.39	2.87	−5.82	−2.96	−6.482	17	< 0.001^*^
Control	Reflex (Raw Score)	−1.06	0.97	−1.56	−0.56	−4.518	16	< 0.001^*^
	Reflex (Standard Score)	−0.71	1.36	−1.40	−0.01	−2.142	16	0.048^*^
	Stationary (Raw Score)	−2.24	1.95	−3.24	−1.23	−4.718	16	< 0.001^*^
	Stationary (Standard Score)	−0.18	0.64	−0.50	0.15	−1.144	16	0.269
	Locomotion (Raw Score)	−5.06	2.36	−6.27	−3.85	−8.847	16	< 0.001^*^
	Locomotion (Standard Score)	−0.71	0.92	−1.18	−0.23	−3.165	16	0.006^*^
	Grasping (Raw Score)	−5.65	2.98	−7.18	−4.12	−7.819	16	< 0.001^*^
	Grasping (Standard Score)	−0.65	0.86	−1.09	−0.20	−3.096	16	0.007^*^
	Visual Motor Integration (Raw Score)	−4.88	2.91	−6.38	−3.39	−6.911	16	< 0.001^*^
	Visual Motor Integration (Standard Score)	−0.29	0.59	−0.60	0.01	−2.063	16	0.056
	GMQ	−1.71	1.11	−2.27	−1.14	−6.366	16	< 0.001^*^
	FMQ	−1.18	1.07	−1.73	−0.62	−4.515	16	< 0.001^*^
	TMQ	−3.19	2.04	−4.28	−2.10	−6.249	15	< 0.001^*^

t: t-value for paired t-test, df: degree of freedom, GMQ: Gross Motor Quotient, FMQ: Fine Motor Quotient, TMQ: Total Motor Quotient,

* statistically significant (level of significance: *p* < 0.05)

The p- and t-values for standard scores in the experimental group were statistically significant for the stationary (t = −2.87, *p* = 0.010), locomotion (t = −3.43, *p* = 0.003), grasp (t = −3.28, *p* = 0.004), and visual motor (t = −5.03, *p* < 0.001) components. P- and t-values in the experimental group were also significant for the GMQ (t = −7.31, *p* < 0.001), TMQ (t = −5.71, *p* < 0.001), FMQ (t = −6.48, *p* < 0.001). The p- and t-values for raw scores in the control group demonstrated significance in the reflex (t = −4.51, *p* < 0.001), stationary (t = −4.71, *p* < 0.001), locomotion (t = −8.84, *p* < 0.001), grasp (t = −7.81, *p* < 0.001), and visual motor (t = −6.91, *p* < 0.001) components. The p- and t-values for standard scores in the control group for reflex (t = −2.14, *p* = 0.048), locomotion (t = −3.16, *p* = 0.006), and grasp (t = −3.09, *p* = 0.007) were also significant. P- and t-values were statistically significant for the GMQ (t = −6.36, *p* < 0.001), FMQ (t = −6.24, *p* < 0.001), and TMQ (t = −4.51, *p* < 0.001) in the control group (within group), and for the GMQ (t = −7.31, *p* < 0.001), FMQ (t = −5.71, *p* < 0.001) and TMQ (t = −6.48, *p* < 0.001) in the experimental group.

The independent sample t-test was applied to obtain results for between-group analysis separately for pre-test and post-test measurements (Table 3). The p- and t-values for raw scores were statistically significant for reflex (t = 3.29, *p* = 0.002) and stationary (t = 4.26, *p* < 0.001) components. The standard scores showed significance only for the stationary component (t = 2.57, *p* = 0.015), and the quotient score was significant for the GMQ (t = 3.275, *p* = 0.002).

**Table 3.** Between-groups comparison of pre- and post-test raw, standard and quotient scores of PDMS-2

**Variable**	**Group**	**Pre**				**Post**			

		**Mean**	**S.D.**	**“t”**	**p value**	**Mean**	**S.D.**	**“t”**	**p value**
Reflex (Raw Score)	Experimental	11.11	1.37	1.033	0.309	12.94	1.11	3.294	0.002^*^
	Control	10.72	0.83			11.82	0.88		
Reflex (Standard Score)	Experimental	11.11	0.90	0.652	0.519	11.56	0.51	0.151	0.881
	Control	10.89	1.13			11.53	0.51		
Stationary (Raw Score)	Experimental	10.44	2.46	1.31	0.199	14.39	1.98	4.263	< 0.001^*^
	Control	9.56	1.50			11.76	1.64		
Stationary (Standard Score)	Experimental	5.39	0.70	0.699	0.489	7.28	3.01	2.573	0.015^*^
	Control	5.22	0.73			5.35	0.70		
Locomotion (Raw Score)	Experimental	8.56	3.07	−0.61	0.546	14.28	2.47	0.46	0.649
	Control	9.06	1.63			13.94	1.78		
Locomotion (Standard Score)	Experimental	5.83	0.86	−0.183	0.856	6.89	0.83	1.674	0.104
	Control	5.89	0.96			6.47	0.62		
Grasping (Raw Score)	Experimental	9.28	3.06	0	1	14.67	2.64	−0.195	0.846
	Control	9.28	1.67			14.82	2.07		
Grasping (Standard Score)	Experimental	6.33	0.49	−0.277	0.783	7.11	0.90	0.404	0.689
	Control	6.39	0.70			7.00	0.71		
Visual Motor Integration (Raw Score)	Experimental	9.00	3.99	−0.454	0.652	14.44	2.23	0.303	0.764
	Control	9.50	2.43			14.24	1.82		
Visual Motor Integration (Standard Score)	Experimental	5.94	0.73	−1.06	0.297	6.83	0.79	1.577	0.124
	Control	6.17	0.51			6.41	0.80		
GMQ	Experimental	22.56	1.46	0.896	0.377	25.44	1.85	3.275	0.002^*^
	Control	22.06	1.86			23.53	1.59		
FMQ	Experimental	12.22	1.06	−1.007	0.321	14.33	1.65	1.288	0.207
	Control	12.56	0.92			13.65	1.50		
TMQ	Experimental	34.61	2.73	0.063	0.95	39.00	2.89	1.668	0.105
	Control	34.56	2.55			37.44	2.53		

t: t-value for independent sample t-test, GMQ: Gross Motor Quotient, FMQ: Fine Motor Quotient, TMQ: Total Motor Quotient,

* statistically significant (level of significance: *p* < 0.05)

## Discussion

The goal of this study was to determine the impact of early play exposure on motor development in high-risk infants using PDMS-2. The experimental group was given a toy kit which had rattles, light and music toys, a water-filled teether, and nesting cups, all of which were appropriate for their age group. Guidance on how to use the toys was also provided. The control group, on the other hand, only received instructions on how to appropriately use play materials that were already available at home.

When the male and female participants in the study were compared, the males outnumbered the females. According to one study, male infants had a much higher neonatal mortality rate than female infants between 2010 and 2018 [21]. A study looking at gender disparities in neonates and early infant mortality also found that males had a higher rate of stillbirths and neonatal deaths [22]. This could be the reason for the discrepancy. Fine motor skills such as grasping and tracking a toy, stationary tasks, and movement was improved in the infants in our study, along with gross motor skills. In one study on the effect of the house setting on infants’ developmental outcomes, the availability of age-appropriate toys and opportunities had a significant impact on the fine motor development and cognition of the child, similarly to in our current study, which also provided age-appropriate play material and activity to improve the infant’s motor development [23].

The intervention group showed higher scores in stationary, locomotion, grasp, and visual motor skills on the PDMS-2 at 7 and 8 months during the post-assessment. This could be linked to the use of toys at a young age, which can aid in the development of developmental aspects, such as in the gross and fine motor, cognitive, and social-emotional domains, all of which contribute to the infant’s overall development. Toys utilised in the intervention were used for kicking and reaching, tummy time, rattling to make sounds, motivating the baby to grasp for objects, and so on. Because there were two follow-ups before the post-assessment, one after two weeks and one after four, the therapist’s regular feedback may have provided motivation to the parents regarding the necessity of early usage of play items. In our study, the experimental group showed a significant improvement in gross motor and fine motor scores, with fine motor scores improving even more since the infants in this group were given age-appropriate play materials such as rattles, light toys, stacking cups, baby teethers, and plush toys. All of these toys have been shown to improve gross motor skills, and especially fine motor skills, because infants have to use their grasp to explore all of the toys. The consequences of physical encounters on later cognitive and social development have been discovered through research, but there has been no evidence for links in studies tying social attention and action skill level to infants’ motor activity or grasping ability. Likewise, early fine and gross motor development affects cognitive capacities such as language and all fine motor activities, which appear to have long-term implications for academic success. The latest findings, which are based on earlier research, imply that an infant’s early motor experiences have a direct impact on their later motor skills. Exploration promotes the growth of physical skills like bilateral and fine motor coordination, nuanced pinch patterns, and even pre-writing abilities [24].

Playing with a toy for an extended period of time may provide additional possibilities for experimenting when it comes to learning about the toy’s properties. Increased imaginative play, which supports expressiveness and affective development, may result from deeper exploration.

The results in the gross motor domain are more significant in the within-group comparison of the control group, because the control group was not given a toy kit, so gross motor was emphasised more than fine motor. The control group’s parents were educated about the early use of age-appropriate toys for their infants, and the importance of each toy was explained on how to use it and what effect it has on the infant. Due to economic constraints, it was revealed that most households possessed few toys, and newborns were exposed to only a few types of play objects. These findings are in line with existing motor learning theories which state that observed performance represents the dynamic interaction between an individual and their environment [25]. Exploration promotes the growth of physical skills like bilateral and fine motor coordination, nuanced pinch patterns, and even pre-writing abilities [24].

The findings of this study reveal that there is no difference in fine or gross motor quotient scores across groups. Since the newborns were not exposed to more play materials and there was less parent-child interaction during play, there is only a difference in the stationary domain of an infant in the control group. The PDMS-2 fine motor components exhibited no improvement.

Play contributes to the formation of Brain-Derived Neurotrophic Factors, a protein that helps present neurons survive while also encouraging the synthesis and division of new neurons and synapses, which is important for improved memory and social interactions. Play aids in the development of long-term memory, cognition, and emotions through facilitating the development of Brain Derived Neurotrophic Factors in the amygdala, frontal cortex, and other brain areas [26]. This type of early intervention has potential effects in terms of boosting neurodevelopmental outcomes, but it comes at a considerable expense. In comparison to previous studies, this is a relatively new and low-cost parent-oriented intervention with the goal of encouraging and supporting positive parenting behaviour. Our findings revealed that introducing high-risk newborns to play materials or toys at an early age can assist them in enhancing their development of motor skills.

## Conclusion

The study looked at the impact of early exposure to play materials on motor performance in high-risk infants, and found that the gross and fine motor components of their development differed significantly. The current study emphasises the importance of early exposure to age-appropriate toys in motor development as a supplement to early intervention for high-risk infants.
